# Comparative Investigation of Coincident Single Nucleotide Polymorphisms Underlying Avian Influenza Viruses in Chickens and Ducks

**DOI:** 10.3390/biology12070969

**Published:** 2023-07-07

**Authors:** Hendrik Bertram, Selina Wilhelmi, Abirami Rajavel, Marc Boelhauve, Margareta Wittmann, Faisal Ramzan, Armin Otto Schmitt, Mehmet Gültas

**Affiliations:** 1 Faculty of Agriculture, South Westphalia University of Applied Sciences, Lübecker Ring 2, 59494 Soest, Germany; hendrik.bertram@stud.uni-goettingen.de (H.B.);; 2 Breeding Informatics Group, Department of Animal Sciences, Georg-August University, Margarethe von Wrangell-Weg 7, 37075 Göttingen, Germany; 3 Center for Integrated Breeding Research (CiBreed), Albrecht-Thaer-Weg 3, Georg-August University, 37075 Göttingen, Germany; 4 Institute of Animal and Dairy Sciences, University of Agriculture, Faisalabad 38000, Pakistan

**Keywords:** single nucleotide polymorphism, coincident SNP, avian influenza, chicken, duck, gene regulation, differentially expressed genes, orthologous variant, downstream, effector, coSNP

## Abstract

**Simple Summary:**

Avian influenza is a serious threat to chickens in comparison to ducks which are more resilient to most virus strains. Today, it is still not completely understood why ducks have more effective inflammatory immune responses. To this extent, the consideration of coincident SNPs (coSNPs) is crucial to unravel genetic programs underlying the susceptibility/resistance of both species to avian influenza. Therefore, our aim is to investigate the differing causal effects of coSNPs on avian-influenza-induced genes, thereby revealing the effector molecules and signaling pathways that mediate an effective immune response after viral infection in ducks and that lead to the disease in chickens.

**Abstract:**

Avian influenza is a severe viral infection that has the potential to cause human pandemics. In particular, chickens are susceptible to many highly pathogenic strains of the virus, resulting in significant losses. In contrast, ducks have been reported to exhibit rapid and effective innate immune responses to most avian influenza virus (AIV) infections. To explore the distinct genetic programs that potentially distinguish the susceptibility/resistance of both species to AIV, the investigation of coincident SNPs (coSNPs) and their differing causal effects on gene functions in both species is important to gain novel insight into the varying immune-related responses of chickens and ducks. By conducting a pairwise genome alignment between these species, we identified coSNPs and their respective effect on AIV-related differentially expressed genes (DEGs) in this study. The examination of these genes (e.g., CD74, RUBCN, and SHTN1 for chickens and ABCA3, MAP2K6, and VIPR2 for ducks) reveals their high relevance to AIV. Further analysis of these genes provides promising effector molecules (such as IκBα, STAT1/STAT3, GSK-3β, or p53) and related key signaling pathways (such as NF-κB, JAK/STAT, or Wnt) to elucidate the complex mechanisms of immune responses to AIV infections in both chickens and ducks.

## 1. Introduction

The awareness of potential zoonotic diseases has recently increased and various strains of avian influenza have had more attention from the general public due to their ongoing effect on the poultry industry. To stress the risks and dangers of avian influenza viruses (AIVs), the World Health Organization closely surveys major events and deaths related to the H5N1 strain of AIV and has reported 868 cases and 457 deaths due to human infections to date [1]. However, the relevance of influenza diseases goes beyond human spread and is most severe in domesticated Galliformes species, such as chickens. According to the latest overview of the European Food Safety Authority, the ongoing European avian influenza epidemic during the influenza seasons of 2021 and 2022 amounted to a total of 2520 outbreak detections of highly pathogenic avian influenza viruses (HPAIVs) in poultry accompanied by 50 million cullings [2]. AIVs belong to the Orthomyxoviridae family of negative-sense RNA viruses. Subtypes, defined by the surface proteins hemagglutinin (H) and neuraminidase (N), notably include H5 and H7 strains that can evolve into highly pathogenic forms in gallinaceous poultry which are able to systemically replicate [3]. In contrast to chickens, waterfowl, and typically wild birds such as mallard ducks, are less prone to most AIV infections and often show no or mild signs of clinical disease due to stronger inflammatory responses. Methods of transmission are primarily via contact with infected birds, their excretions, or through contaminated water and feed. Immune responses also vary between species. For example, rapid apoptosis induction during HPAIV infection in ducks and delayed induction in chickens can influence the efficiency of viral infection control [4].

In recent decades, researchers have, therefore, made an extensive effort towards understanding AIV disease progression in chickens and ducks to investigate the causes of disease susceptibility and resistance. A considerable number of comparative analyses regarding the genetic repertoire, gene functions, and evolutionary selection related to immune responses in different bird species, including chickens and ducks, have been performed and published [5,6,7,8,9,10,11,12,13,14,15,16,17,18,19,20,21]. In this regard, pattern recognition receptors (PRRs) have been put in the spotlight due to their rapid initiation of immune responses to pathogens such as AIVs [7,10,22]. In particular, the susceptibility of chickens can be partly explained by their lack of the retinoic acid-inducible gene I (RIG-I) and the RIG-I binding protein RNF135 [7,9,11,22,23,24,25,26,27]. However, the absence of RIG-I in chickens is partly compensated for by the RIG-I-like receptor (RLR) MDA5 which is adapted in chickens to target small dsRNA [9,23,24,27]. In an effort to comprehend the underlying gene activities and pathways induced by AIVs, several studies conducted gene expression analyses in infected chickens and ducks [3,9,10,11,12,13,14,15,23,24,25,26,27,28,29,30,31,32,33,34,35,36,37,38,39,40,41]. While the host–pathogen interaction of AIVs is highly complex and nuanced, differing between pathogen and species, recent advances have shed some light on the mechanisms behind the immune responses of chickens and ducks [22]. For this purpose, Ranaware et al. [28] showcased the relation of disease severity to a strong up-regulation of gene expression of interferons (IFNs), pro-inflammatory cytokines and chemokines as well as IFN-stimulated genes (ISGs) during H5N1 HPAIV infection in lung in chickens and observed a lack of this expression during low pathogenic AIV (LPAIV) H9N2 infection. To further emphasize the complex transcriptomic signature, Wang et al. [34] observed high expression of miRNAs and moderate gene expression during LPAIV H5N3 infection in lung tissue of broilers and also highlighted the importance of miRNA for the regulation of genes. In contrast to chickens, ducks typically induce effective inflammatory responses to most AIV strains. This is demonstrated by the findings of Campbell et al. [41], who studied the tissue-specific responses of ducks during HPAIV H5N1 and LPAIV H5N2 infection, in which robust and high gene expression was observed. Specifically, they highlight the strong early response of PRRs and ISGs in duck tissues combined with a decrease in expression of proinflammatory cytokines in the lung and key components of leukocyte recruitment and complement pathways in the intestine [41]. For comprehensive overviews of differential gene expression and innate immunity related to AIV infection in chickens and ducks, we refer to [6,7,8,22].

While the majority of studies on AIVs have focused on either chickens or ducks, there were also experiments conducted on both bird species under the same conditions that permit their direct comparison [3,42,43,44,45,46]. For example, Cornelissen et al. [46] discussed the innate immune responses of LPAIV H7N1 in chickens and ducks and highlighted differences in gene expression such as the observation of a correlation of Toll-like receptor 7 and MDA5 responses to IFN-α and IFN-β in chickens and its absence in ducks. Other studies focused on exploring immune-related genes during HPAIV infection [42,43,44,45]. Hu et al. [43] demonstrated that PA-X decreases virulence of H5N1 by inhibiting viral replication and observed stronger expression of cytokines in the hearts and brains of ducks than in lungs and spleens, whereas the expression was evenly spread in chickens. Another related study was conducted by Smith et al. [3] who performed transcriptome analysis and inspected the relevance of IFN-induced transmembrane proteins and their evolutionary selection for AIV defense in chickens and ducks. Continuing on the study by Smith et al. [3], Klees et al. [47] further investigated the dataset and conducted upstream analysis on the differentially expressed genes (DEGs) with a focus on the effective immune response of ducks during HPAIV infection. The findings of Klees et al. [47] put an emphasis on the regulatory motifs that control the gene expression in ducks to curb disease progression and failed to achieve this in chickens. Among other regulatory factors, the importance of EGR1, FOS, SRF, SP1, EP300, RUNX2, MYC, SMAD3, SMAD4, and ETS1 were discussed for regulatory processes in chickens and ducks.

**Figure 1:**
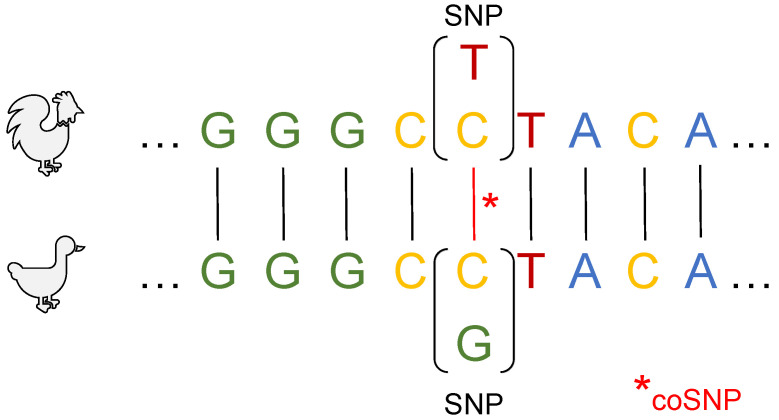
An illustration of a coincident SNP (coSNP) in chickens and ducks. Reference sequences from chickens and ducks are arranged in a sequence alignment block. The centre position of this alignment, highlighted by (*), shows the occurrence of a coSNP, which is localized at the orthologous position.

Another fundamental aspect to systematically explore genetic programs during AIV progression in both species is the consideration of coincident single nucleotide polymorphisms (coSNPs) that occur at orthologous positions [48]. Due to their orthologous positions in the genomes of species, coSNPs should presumably have a similar impact on genes or their protein products, e.g., being disruptive or non-disruptive. An illustration of the context of coSNPs in chickens and ducks is shown in Figure 1. While a variety of studies have been published using coSNPs to study evolutionary processes [48,49,50,51,52,53,54,55], recent studies employed coSNPs to identify genetic mechanisms related to functional variants across different species including humans, chimpanzees or livestock [56,57,58]. Given the importance and availability of SNP markers in breeding, investigating coSNPs and their differing causal impacts on gene functions could be promising for gaining greater insight into disease resistance or susceptibility and finding suitable genetic targets.

Despite a rich literature on AIV progression in chickens and ducks, the importance and potential of coSNPs for cross-species comparisons to decipher the different immune-related functions underlying AIV infection in both species has not yet been extensively studied. To address the limited knowledge available on the causal effects of coSNPs in association with AIV progression in chickens and ducks, we aim to analyze the biological functions of the related genes harboring coSNPs for the identification of affected downstream pathways and effector molecules in this study. We initially conducted pairwise genome alignments to identify orthologous regions, which were subsequently used for the determination of the coSNPs. By applying the *SnpEff* tool [59], the potential impact of coSNPs on gene functions was examined in chickens and ducks. Based on the results, we generated lists of candidate genes by incorporating their different expression patterns during AIV infection for each species. Finally, we revealed the relevant downstream effectors that significantly influence the activation and regulation of various downstream signaling pathways. These findings could be promising to elucidate the complex mechanisms of immune responses to AIV infections in both chicken and duck populations and to further design novel hypotheses and potential targets for breeding objectives as well as therapeutic strategies.

## 2. Materials and Methods

**Figure 2:**
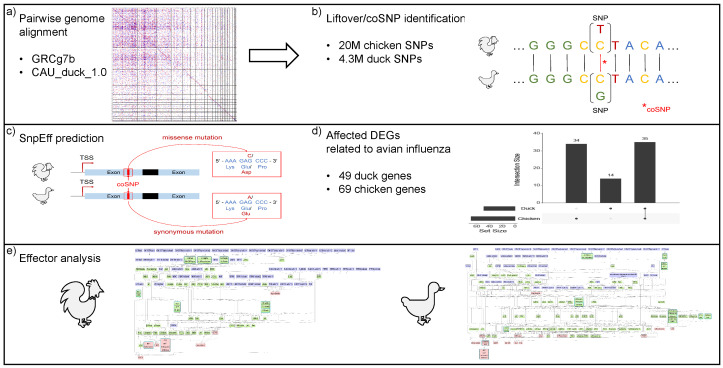
Overview of the workflow applied in this study. First, (**a**) the chicken and duck genomes are aligned and (**b**) orthologous variants (coSNPs) are identified. Second, (**c**) the effect of coSNPs on genes is predicted using *SnpEff* [59] and (**d**) filtered for differentially expressed genes (DEGs) during avian influenza virus (AIV) infection. Lastly, (**e**) downstream effectors are predicted based on the involved signaling pathways.

In this section, we describe the data set analyzed and methods applied, starting with the identification of coSNPs and followed by an SNP effect prediction and a downstream analysis of the affected genes. An overview of our analysis workflow is given in Figure 2.

The implementation of the coSNP identification pipeline and the subsequent analysis steps are provided as python and R scripts in Supplementary File S1 to guarantee reproducibility.

### 2.1. Genome Data

In our study, we incorporated publicly available genomic data for chickens and ducks, including gene and variant annotations for the assemblies *GRCg7b*, *CAU_duck1.0*, and *BGI_duck_1.0*, which are provided by Ensembl release 109 [60]. Duck SNPs were obtained from Genome Variation Map [61] annotated to the *BGI_duck_1.0* duck assembly due to the sparsity of public collections of variants in ducks.

### 2.2. Identification of coSNPs

To identify coSNPs, localized at orthologous genomic positions of chickens and ducks, we followed the methodology of Zhao et al. [57]. Our analysis pipeline consists of the following three steps:

- **Lift over**: Following the *nf-LO* workflow by Talenti and Prendergast [62], we aligned the genomes *BGI_duck_1.0* and *CAU_duck_1.0* with the *LAST* alignment program [63] where fitting alignment parameters were estimated by the *last-train* program [64]. Afterward, bijective chain files, e.g., unique alignment blocks in both genome versions, were created and assembled to nets with *axtChain*, *chainNet* and *netChainSubset* from UCSC [62,65]. Finally, SNP annotations were lifted over by using *CrossMap* [66] to obtain remapped SNP annotations for the *CAU_duck_1.0* genome. Although the variant data and genome annotations of chickens have been annotated to the same genome (*GRCg7b*), it is noteworthy that the genomic coordinates for SNP data of ducks had to be lifted over from *BGI_duck_1.0* (a scaffold assembly) to *CAU_duck1.0* (chromosome-level assembly) to fit the preceding DEG annotations.
- **coSNP localization**: The same lift-over procedure was repeated for discovering orthologous positions between the chicken genome *GRCg7b* and the duck genome *CAU_duck1.0*. The resulting positional and chromosomal information was used to localize genome-wide coSNPs (as illustrated in Figure 1). In summary, using a total of 4,393,763 duck and 20,066,289 chicken SNPs, we were able to localize 84,898 coSNPs of which 37,242 were located in 7387 unique duck and 7398 unique chicken genes. The list of identified coSNPs is given in Supplementary Table S1.
- **SNP effect prediction:** We have applied the *SnpEff* tool [59] considering the databases of the latest Ensembl release (109) [60] to predict the potential impact of coSNPs on gene functions in chickens and ducks, respectively. For this analysis step, we manually built the *SnpEff* database using the *build* command for the chicken genome *GRCg7b* and the duck genome *CAU_duck1.0* and their respective *gtf* gene annotations. The genome-wide functional classification of coSNPs by *SnpEff* includes: (i) low impact variants such as synonymous variants; (ii) moderate impact variants such as non-disruptive variants that can change protein effectiveness; (iii) high impact variants such as disruptive variants that can cause loss of function [67]. The consequences of coSNPs on the genes and their transcripts in both bird species are likely to provide valuable insight into understanding the disease progression of avian influenza. Among all the genes in chickens and ducks that contain a coSNP, 390 and 469 genes were impacted by at least one moderate or high consequence, respectively. A detailed overview of the coSNP consequences is given in Supplementary Table S2.

### 2.3. Identification of Candidate Genes

To focus on the potentially divergent effect of coSNPs on downstream mechanisms in chickens and ducks, we consider the transcriptome data of Smith et al. [3] sampled during avian influenza disease progression. The related experiments were conducted on 20 white leghorn chickens and 20 domestic gray mallards focusing on the differences in the gene expression response to HPAIV H5N1 and LPAIV H5N2 by considering lung and ileum tissues sampled at one and three days post-infection (dpi) including mock infections and three biological replicates for each condition. Klees et al. [47] used this data set to identify DEGs between the experimental and control groups across two tissues in both chickens and ducks by setting the significance thresholds as |$log_{2}$ fold change| > 0.58 and the false discovery rate (FDR) adjusted *p*-value < 0.05. They further identified the upstream regulators through a comparative assessment of disease progression. For more information on the experimental design and data analysis of DEGs, we refer to the original study by Smith et al. [3] and the study by Klees et al. [47].

To construct our candidate gene lists, a list of 2345 DEGs was obtained from Klees et al. [47] where all DEGs from all experimental conditions in ducks were merged into a large set of regulated genes as a response to AIV infection (see Supplementary Table S3). We assume that the regulation and involved pathways of these DEGs induce a rapid and effective response in ducks because we observed a lack of DEGs in almost all experimental conditions in chickens. This sparsity of DEGs in chickens during AIV infection was already discussed by Klees et al. [47]. Therefore, we only considered DEGs in ducks and their orthologous genes in chickens for our comparison. Analogous to Klees et al. [47], orthologs were retrieved from BioMart web services [68]. Finally, following the methodology by Vijayakumar et al. [67], we filtered our candidate gene lists in chickens and ducks separately based on a moderate or high impact classification predicted by *SnpEff* [59]. Thus, the final gene lists for chickens and ducks contain 69 and 49 genes, respectively, of which 35 orthologs are common. The gene lists are given in Supplementary Files S2 and S3.

### 2.4. Downstream Effector Analysis

To gain more insight into the functional role of candidate genes in the initiation, modulation, and resolution of immune responses to AIVs, we identified key signaling pathways and effector molecules using candidate genes for both chickens and ducks. Effectors are crucial signaling molecules that act as end products located several steps downstream and regulate the function of numerous signaling cascades [69]. In the context of AIVs, exploring and understanding the role of effectors in association with coSNPs and their corresponding candidate genes could provide important knowledge for unraveling effective or perturbed immune responses. Similar to the previous study by Rajavel et al. [69], we performed an effector molecule search for both the chicken and the duck gene list on the bioinformatics platform geneXplain [70] using the TRANSPATH ^®^ database [71]. Recommended parameters of the effector search workflow were set according to Rajavel et al. [69] with a maximum search radius of 10, a FDR adjusted *p*-value <0.05 and a *z*-score >1.0. Furthermore, we employed a pathway enrichment search using the Reactome database [72] and an FDR adjusted *p*-value <0.05 to check if a pathway is mainly affected by our genes.

## 3. Results and Discussion

In this study, we conducted an investigation into the effects of coSNPs by analyzing their causal impact on genes associated with resistance or susceptibility to AIV infection in both chickens and ducks. Our methodology involved performing pairwise genome alignments for both species and identifying the orthologous genomic regions containing coSNPs. These regions were then evaluated for their potential impact on gene functions using the *SnpEff* tool [59]. Subsequently, we generated a list of candidate genes for each species, which were filtered based on their differential gene expression patterns in lung and ileum tissues during AIV infection. The corresponding downstream pathways and effectors of the candidate genes affected by coSNPs might be promising targets that restrict avian influenza disease progression in ducks or alleviate disease progression in chickens.

This section is split into four parts. The first part explores and highlights the different effects of coSNPs to gain insight into the candidate gene lists. In the second part, we briefly present an overview of the results of the downstream analysis. In the third part, we contrast the enriched pathways of chickens and ducks to elaborate on the background of the affected genes. Finally, we performed a downstream effector analysis to identify the most important molecules that act in the downstream signaling cascades which might be affected by coSNPs.

### 3.1. Effects of coSNPs

In our analysis, we identified 49 candidate genes in ducks and 69 candidate genes in chickens, with 35 genes being common to both species. This substantial overlap might suggest that coSNPs have a comparable impact on genes in both bird species. Interestingly, our investigation revealed a set of candidate genes exhibiting distinct impact patterns in chickens and ducks that are caused by coSNPs. A small number of notable genes related to AIV infection are exemplarily illustrated in Table 1. Comprehensive summaries of all coSNP-induced impacts on both gene lists are available in Supplementary Tables S4 and S5.

In our study, we identified several coSNPs that lead to missense mutations in chickens or ducks which might affect the stability, structure, or function of essential proteins involved in AIV defense. Among the genes given in Table 1, coSNPs in the CD74 and Rubicon (RUBCN) genes lead to a missense mutation in chickens. The CD74 gene is an important gene that performs diverse functions in the innate immune response. It encodes an essential chaperone that modulates antigen presentation in response to the class II major histocompatibility complex and additionally plays crucial roles as a receptor on the cell surface for the cytokine macrophage migration inhibitory factor or in the interaction with the amyloid precursor protein [73]. Interestingly, previous studies have reported a decrease in CD74 expression related to AIV infection. Ibaez et al. [74] identified a decrease in CD74 expression in the alveolar epithelial cells in mice during H1N1 and H3N2 infection. In line with this, the findings of Xing et al. [75] show a down-regulation of CD74 during H9N2 and H6N2 LPAIV infection in the lungs of chickens. On the other hand, in ducks, this mutation is located in an intron of the recently identified gene, ENSAPLG00000013923, presumably encoding the ribosomal protein RPS14, which is an integral component of the small ribosomal subunit (40S) and plays a crucial role in protein biosynthesis. To shed further light on the expression patterns of the candidate genes, we also examined the underlying transcriptome data of Smith et al. [3]. Our observations revealed that ENSAPLG00000013923 was down-regulated in the lung during H5N1 infection at 1 dpi. Another interesting gene is RUBCN, which encodes a protein that functions as a negative regulator of autophagy and endocytic trafficking and also plays an important role in controlling endosome maturation [76]. Moreover, RUBCN has the ability to target various signaling complexes, therefore coordinating immune responses. Specifically, RUBCN can act as a feedback inhibitor to prevent unbalanced proinflammatory responses through the mediation of RLR signaling, and its expression has been shown to impact AIV host defense in mouse [77]. A closer look at the expression values reveals that RUBCN was up-regulated in the lung at 1 dpi during H5N1 infection, indicating its role in early pro-inflammatory response reduction in ducks.

Furthermore, the identification of coSNPs within the candidate genes ABCA3, MAP2K6, and VIPR2 (also known as RSAD2) has exposed missense mutations in ducks. The ABCA3 gene belongs to the ABC transporter family and is involved in maintaining cellular cholesterol and phospholipid homeostasis [78]. Interestingly, a paralog of ABCA3, ABCA1, was shown to be associated with HPAIV survival in chickens [17]. Our data supports the importance of ABCA3 which was up-regulated in the lungs of ducks at 3 dpi during H5N1 HPAIV infection. The MAP2K6 gene is known for its critical involvement in the MAPK signaling pathway which has been implicated in modulating the type I IFN response [28,29], and has also been linked to the survival of chickens infected with HPAIV [16,17]. The differential expression of MAPK-related genes during HPAIV infection has been observed in chicken [28,29], while they were down-regulated in duck endothelial cells [79]. In line with these findings, we report the down-regulation of MAP2K6 in duck ileum at 3 dpi during H5N1 infection. The third gene, VIPR2 has a crucial function in antiviral defense. It is stimulated by type I IFN and is acknowledged as an inhibitor of AIVs via the suppression of viral budding [80]. Prior research indicates substantial up-regulation of VIPR2 in various chicken and duck tissues during H5N1 infection [26], a finding that is consistent with the results of this study, where VIPR2 was found to be up-regulated during H5N1 infection in duck lung at 1 dpi.

While missense mutations can have a severe effect on proteins, in the following, we want to discuss coSNPs that lead to particularly detrimental outcomes on AIV-related genes in chickens and ducks. One of the genes of interest is the chicken gene ENSGALG00010000247 and its orthologous duck gene, ENSAPLG00000008507, which are novel gene predictions and are affected by a coSNP that presumably causes a premature stop gain. Stop gain variants, commonly known as nonsense mutations, introduce a premature stop codon to a gene and can lead to truncated protein products that lose their function and decrease overall fitness if they are not cleared by nonsense-mediated decay pathways. Considering the transcriptome data, ENSAPLG00000008507 was up-regulated during H5N1 infection in the ileum at 1 dpi.

Another substantial impact is caused by coSNPs that occur within either acceptor or donor splice sites of the genes Shootin 1 (SHTN1) in chickens as well as ATP8A1 and ENSAPLG00000022349 in ducks, as shown in Table 1. Remarkably, mutations in the splicing apparatus may have a significant impact on gene transcripts by means of alternative splicing, potentially producing a novel protein isoform, and are suggested to explain parts of phenotypic variability of diseases [81]. In line with this, various antiviral genes have been reported to be modulated by alternative splicing or splicing variants, which may either promote or impair their antiviral potency [82,83]. Among the genes that affect splicing, SHTN1 is involved in the positive regulation of neuron migration, as well as the regulation of signaling pathways such as CDC42, RAC1, and PAK1, and the regulation of phosphoinositide 3-kinase (PI3K) activity [84]. Notably, PAK1 activation has been shown to contribute to AIV replication in human lung epithelial cells [85]. Furthermore, the associated gene PI3K, as well as its downstream pathway involving Akt, are implicated to have an ambivalent role in AIV replication, as they can both support viral replication and co-activate antiviral responses [86]. However, inhibition of PI3K has been suggested to reduce viral titers of some AIV strains [87]. In addition, we also identified two coSNPs that may lead to aberrant proteins in ducks, namely a potentially defective splice donor site in ATP8A1 as well as a potentially defective splice acceptor site in the duck gene ENSAPLG00000022349 where the orthologous coSNP is located in an intron of the chicken gene ERF3B. ATP8A1 is a p-type ATPase that participates in various innate immunity-related processes, such as cell migration and transportation. In particular, P4-ATPases such as ATP8A1, along with CDC50 family members, form a phospholipid flippase complex which is involved in the translocation of aminophospholipids such as phosphatidylserine from the outer to the inner leaflet of membranes [88]. Interestingly, phospholipids, such as phosphatidylserine, are components of the cell membrane and are involved in various signaling processes such as cell cycle signaling for apoptosis, an important process in killing infected cells. While the function of ENSAPLG00000022349 remains unknown, it is likely to be an ortholog of the human tripartite motif containing 35 genes (TRIM35) based on Ensembl. TRIM proteins are known to induce E3 ubiquitin ligase activity and have been implicated in downstream processes of the innate immune response in mammals [22,89,90]. Notably, TRIM25 has been identified as an important molecule in the innate immunity of both chickens and ducks against AIVs, interacting with RLR signaling [25,26]. Furthermore, TRIM35 has been demonstrated to positively regulate RIG-I mediated signaling by promoting TRAF3 activation during AIV infection in mouse [91], indicating its potential as an interesting protein to investigate for AIV resistance in chickens and ducks. On the other hand, ERF3B encodes a GTPase that belongs to the GTP-binding elongation factor family, is involved in translation termination, and may play a role in mRNA stability and cell cycle progression [92]. Our transcriptome data revealed the down-regulation of ATP8A1 in the ileum in ducks during H5N1 infection at 3 dpi as well as the up-regulation of SHTN1 and ENSAPLG00000022349 in the lung at 3 dpi and 1 dpi, respectively.

Overall, the knowledge of coSNPs and the investigation of genes harboring them provide crucial insights into their potential impact on susceptibility or resistance to AIVs, thereby aiding in the differentiation of underlying genetic mechanisms in chickens and ducks.

### 3.2. Identification of Pathways and Downstream Effectors

Based on the candidate gene lists, we performed the effector search algorithm and a pathway enrichment analysis, for the identification of downstream molecules as well as pathways that are targeted by the genes of interest. Lists of the enriched pathways and key downstream effectors are presented in Table 2 and Table 3. Detailed results of effector and pathway analyses are given in Supplementary Tables S6 and S7 as well as Tables S8 and S9, respectively. Despite a high degree of overlap between the gene lists of both species, Table 2 and Table 3 reveal substantial differences in the effectors and enriched pathways, indicating considerable variability in their respective numbers of detections. Notably, while the number of candidate genes in ducks is smaller than in chickens, the former exhibits a greater number of effectors.

### 3.3. Pathway Analysis

Pathway enrichment analysis of the candidate genes has revealed one pathway for chickens and four pathways for ducks, as shown in Table 2.

Notably, we identified a pathway linked to interleukins (ILs) IL-3 and IL-5 and the granulocyte-macrophage colony-stimulating factor (GM-CSF) in chickens, whereas several pathways associated with IFN signaling were found in ducks. Despite both ILs and IFNs belonging to the group of cytokines, their roles in viral response vary considerably. IFNs are the principal regulators of innate immunity, and the early induction of the type I IFN response is deemed an essential factor for the effective immune response of duck following AIV infection [37,93]. In this context, the antiviral activities and apoptosis induction of IFN-α and IFN-β were extensively investigated [94,95]. In contrast to IFNs, the multifaceted functions of ILs can promote as well as inhibit inflammation [93]. In this regard, IL-3 and IL-5, together with GM-CSF, are pleiotropic regulators of inflammation that play a role in the rapid clearance of pathogens [96]. GM-CSF is a glycoprotein that enhances antigen presentation, microbicidal capacity, and leukocyte chemotaxis, and is critical in the homeostasis process of pulmonary alveolar macrophages, which, when deficient, can result in pulmonary alveolar proteinosis [97]. Due to its crucial dual role in both inflammatory responses and the development of chronic inflammation [96], GM-CSF might be an attractive therapeutic target, which has already been applied as a vaccine adjuvant against viral infections [98,99,100,101].

In line with the findings described in Section 3.1 and the identified effectors (see Section 3.4), IL-3, IL-5, and GM-CSF are known to activate the JAK/STAT, Ras/MAPK, and PI3K pathways [102]. The presence of several ILs at the site of inflammation characterizes pathological cytokine and chemokine overproduction, commonly referred to as a “cytokine storm” [93,103]. However, since our data set did not capture the early response and due to the limited gene expression at 1 dpi or 3 dpi, there is currently no conclusive evidence of cytokine overexpression in chickens.

### 3.4. Downstream Effector Analysis

Our analysis of the candidate gene lists identified seven key downstream effectors in chickens and 15 key downstream effectors in ducks, as shown in Table 3. Among the effectors, IκBα, STAT1, and STAT3 were common to both bird species. Additionally, there was an overlap between some effectors associated with APC, axin1, β-catenin, and GSK-3β in terms of protein complexes. The notation used, e.g., axin1:β-catenin, denotes a protein complex or interaction between axin1 and β-catenin. In the following sections, we present the main results explaining the functions of these effectors in the context of AIV infection.

#### 3.4.1. Downstream Effectors in Chicken

**Figure 3:**
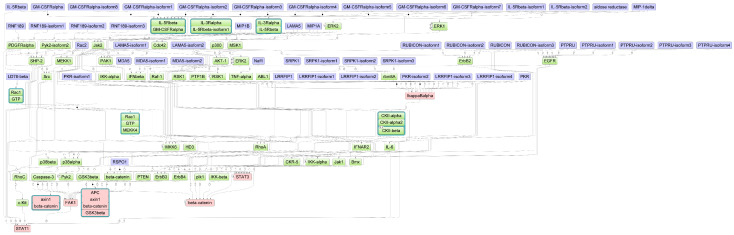
Downstream effectors and their interactions identified in chickens. Blue nodes denote the candidate genes, green nodes indicate intermediate interaction partners and red nodes represent the identified downstream effectors. A high-resolution version of this image is provided in Supplementary Figure S1.

Three proteins and protein complexes related to the Wnt signaling pathway, including axin1:β-catenin, β-catenin, and APC:axin1:β-catenin-GSK-3β were identified as effectors in chickens (Figure 3). In addition, other effectors in chickens were related to NF-κB (IκBα) as well as IFN and JAK/STAT signaling (STAT1 and STAT3).

The only chicken-specific effector is the focal adhesion kinase 1 (FAK1), which is involved in pathways related to apoptosis, cellular adhesion, and cell migration. It has been shown that FAK regulates AIV entry at a post-internalization step and its inhibition reduces AIV polymerase activity in vitro in human lung and bronchial cells [104]. The activity of FAK is suggested to be required for the nuclear localization of NF-κB during AIV infection in mice [105]. Since HPAIVs hijack important anti-viral related pathways to promote their replication, proper governance or inhibition of FAK and NF-κB could be prime targets for the viral susceptibility of chickens. This claim is supported by findings of Perlas et al. [106], who observed the up-regulation of NF-κB-related genes (PLAU, VCAM1, TNFRSF1A) and MAPK-related genes (TNFRSF1, PGF) in chicken breeds susceptible to HPAIV strains, whereas they were found down-regulated in more resistant chicken breeds. In line with this, Drobik-Czwarno et al. [17] identified the NF-κB-related gene TNFRSF1A in a region associated with HPAIV survival of chickens.

#### 3.4.2. Downstream Effectors in Duck

**Figure 4:**
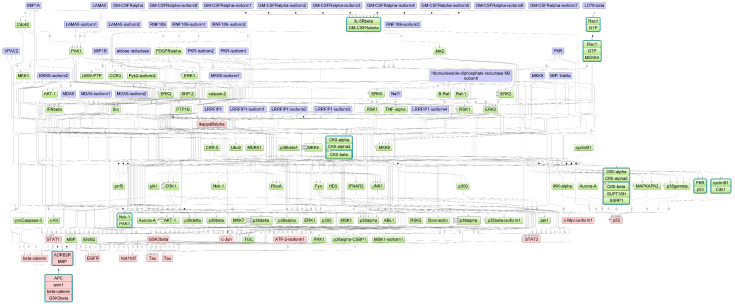
Downstream effectors and their interactions identified in ducks. Blue nodes denote the candidate genes, green nodes indicate intermediate interaction partners and red nodes represent the identified downstream effectors. A high-resolution version of this image is provided in Supplementary Figure S2.

In comparison to chickens, we identified a set of eight duck-specific effectors (Figure 4). A closer look at these effectors shows a diversity of complex functional features in ducks in response to AIV infection. Some of the effectors have direct relevance for AIV, while the others could be indirectly linked via interactions.

In particular, p53, c-Jun, ATF-2, EGFR, and c-Myc are known for their important roles in various immune functions. The effector p53 is known as the “guardian of the genome” [107] which is a tumor suppressor gene that is activated indirectly by type I IFN and its function is crucial in responding to viral infections through p53-dependent apoptosis [95]. In this regard, p53 plays an important role in innate immunity by boosting IFN-dependent antiviral responses [108]. In mouse models, it was reported that the absence of p53 responses results in increased susceptibility to viral infections [109]. Furthermore, the proto-oncogene c-Jun is a member of the AP-1 transcription factor (TF) family and plays a vital role in regulating cell proliferation, differentiation, and apoptosis. Remarkably, c-Jun and its related c-Jun N-terminal kinases (JNKs), which bind and phosphorylate c-Jun, respond to stress stimuli and are involved in regulating various host immune responses and genes such as PI3K, ATF-2, SMAD4, p53, STAT3, and GM-CSF [110]. In this context, the down-regulation of c-Jun was found to suppress H5N1 viral replication in vitro in human cells and in vivo in mouse [111]. Along with c-Jun and NF-κB, the effector ATF-2 has been reported to induce the production of type I IFN, which is well-studied in the generation of antiviral responses by up-regulating genes that activate dendritic cells and natural killer cells, both of which are involved in adaptive immunity [112].

Furthermore, EGFR is a transmembrane glycoprotein that is a member of the protein kinase superfamily. It is a receptor for the epidermal growth factor that induces cell proliferation and acts in downstream signal transduction, activating several proteins related to Ras/MAPK, Akt, and JNK pathways [113]. Inhibition of EGFR has been found to decrease viral uptake in human lung epithelial cells by activating PI3K [114], suggesting that EGFR might be a potential therapeutic target for chicken and duck disease progression caused by AIVs. Another well-studied effector in cancer research is c-Myc, which encodes a proto-oncogenic nuclear phosphoprotein and participates in various cellular processes such as cell cycle progression, apoptosis, and cellular transformation and directly or indirectly interacts with a variety of pathways such as NF-κB or Wnt and EGF via MAPK/ERK [115,116]. Of note, the mRNA of c-Myc contains an internal ribosome entry site that enables RNA translation even when 5’cap dependent translation is inhibited, such as under stress responses to viral infections [117]. Therefore, in addition to its role in cancer, c-Myc may represent an intriguing target for further investigation in AIV infection due to its involvement in cellular apoptosis during stress responses.

The remaining effectors ADRB2R:MBP, Tau, and Hist1h3f have been implicated in various biological processes but their direct association with immune responses is unknown. For example, the effector ADRB2R encodes the beta-2 adrenergic receptor, which mediates many aspects of airway function in humans, is expressed by most immune cells, and is mainly found in the lung [118]. On the other hand, the effectors MBP and Tau are involved in the biological processes linked to the nervous system [119,120]. The Hist1h3f is another effector which encodes the H3 clustered histone 1 and plays a crucial role in transcriptional regulation, DNA replication, DNA repair, and chromosomal stability. Due to their involvement in chromatin remodeling, histones may also play a crucial role in regulating epigenetic processes in host defense against viral infections by controlling the DNA permissibility for rapid and directed changes in gene expression [121]. In agreement with this, Hoeksema et al. [122] have shown that histones H4 and H3 inhibit the infectivity of some influenza strains in human lung epithelial cells. Taken together, these effectors could be promising targets for future studies due to their functional roles, such as (i) context-dependent production of proinflammatory cytokines of ADRB2R [118]; (ii) involvement in the signaling of MBP; (iii) association of Tau with target genes related to viral infection such as GSK-3β, CDK5, and JNK [120]; (iv) controlling DNA permissibility to rapidly direct viral gene expression of Hist1h3f [121,122].

#### 3.4.3. Common Effectors

Due to the shared pool of affected genes, several effectors intersect the downstream signaling of both chickens and ducks. IκBα is among the common effectors in both chickens and ducks. It encodes a potent inhibitor of NF-κB, a TF that plays a pivotal role in regulating various pathways, including immune responses to infections, where it controls the expression of inflammatory cytokines [123]. Previous studies showed that even though NF-κB acts as a master regulator of the innate immune defense and induces antiviral activities, AIVs also depend on NF-κB where viruses can employ the functions of NF-κB to increase viral replication [124,125]. In line with this, IκBα was also found to be associated with HPAIV resistance in chicken [17] and hence might play a crucial role in the delicate control of NF-κB in both chickens and ducks to prevent AIVs from exploiting the related pathways to their benefit.

Interestingly, we identified two members of the signal transducer and activator of the transcription family (STAT), STAT1 and STAT3, as common effectors in both chickens and ducks. Due to their prominent role in the regulation of the IFN response via immune signaling pathways such as JAK/STAT, STAT TFs are well-studied and there is rich literature available for their related pathways. For example, Kuchipudi et al. [42] reported the inhibition and down-regulation of STAT3 in chickens, while the expression of STAT3 in ducks was unaffected and up-regulated. Similarly, Jia et al. [126] showed that the viral NS1 gene interferes with STAT members and the IFN signaling to increase viral replication in vitro. Klees et al. [47] discussed the importance of STAT TFs as upstream regulators of the DEGs following AIV infection, which induces type I IFN response and ISGs [127]. In this regard, STAT members (STAT1, STAT3, and STAT4) were up-regulated during H5N1 infection in ducks, but there was no response in chickens. Additionally, Klees et al. [47] determined that STAT TF binding sites were only enriched in the promoters of the duck DEGs and not in orthologous chicken promoters. Therefore, our findings of effectors suggest that STAT members play an essential role in both bird species for viral defense, but pathways in chickens may be compromised or inhibited by AIVs due to a lack of enriched STAT binding sites and dysregulated pathways.

The identified effectors in the final group are associated with the protein complexes of APC, axin1, β-catenin, and GSK-3β, which are crucial components of the Wnt signaling pathways. GSK-3β, a serine-threonine kinase from the glycogen synthase kinase family, negatively regulates glucose homeostasis and is at the crosspoint between a multitude of pathways related to functions such as cellular signaling or regulation of inflammation [128,129]. Many pathways, such as mTOR, PI3K/Akt, and Ras/MAPK, target GSK-3 inhibition to promote dephosphorylation of GSK-3 substrates, including c-Myc or c-Jun, which could stimulate cell proliferation, migration, and survival [128]. A recent study highlighted the regulatory role of GSK-3β, along with β-catenin, in controlling the antiviral innate immune response to viral infections, causing a rapid induction of type I IFN response and activation of IFN regulatory factor 3 [130]. In the context of Wnt signaling modulation, among other proteins, GSK-3, axin, and APC are involved in the degradation of β-catenin [131]. Furthermore, the targeted Wnt signaling pathway is intertwined with various pathways, including the TGF-β pathway, where SMADs and β-catenin can interact via the lymphoid enhancer-binding factor and T-cell-specific TF [132]. By considering duck DEGs and their orthologs in chickens, Klees et al. [47] reported the enrichment of SMAD members in the promoters of chicken genes. However, SMAD members only acted as upstream master regulators of duck DEGs during AIV infection. These findings demonstrate the complex interplay and entanglement of pathways such as Wnt in the immune response, emphasizing the importance of their orchestration via SMAD members.

## 4. Conclusions

Despite the rapid progress in genomics and transcriptomics, the nuanced differences underlying susceptibility or resistance to avian influenza in chickens and ducks remain incompletely understood. To decipher the distinct genetic programs underlying immune-related functions, which could potentially distinguish the susceptibility/resistance of both species to avian influenza, the consideration of coSNPs is essential in the determination of novel targets in breeding research. Hence, we employed a genome-wide analysis to identify coSNPs and their differing causal impacts on AIV-related candidate genes. To this end, downstream pathway and effector analyses were examined that are likely linked to the divergent immune responses in both species. Our findings demonstrate that although the impact patterns of coSNPs on affected genes are mostly similar between chickens and ducks, we discovered several promising coSNPs that have divergent effects on genes in chickens (e.g., CD74, RUBCN, SHTN1, and ENSGALG00010000247) and ducks (e.g., ABCA3, MAP2K6, VIPR2, ATP8A1, and ENSAPLG00000022349) corresponding to immune responses induced by AIVs. Moreover, the downstream pathway and effector analysis reveal species-specific and commonly targeted biological mechanisms, which might partially explain the effective or ineffective immune responses of both species. In particular, we report that the candidate genes might be linked to IL signaling pathways in chickens and IFN and immune signaling pathways in ducks. On top of that, our study extends the previous transcriptome analysis by Smith et al. [3] and upstream analysis by Klees et al. [47] by leveraging different causal impacts of coSNPs on gene functions of both species to comparatively explore their downstream mechanisms. To the best of our knowledge, this is the first study that considers coSNPs in the context of AIV infection, offering a basis for the formulation of novel hypotheses in future research.

## Figures and Tables

**Table 1:** Highlight of effects of coSNPs in candidate genes in chickens and ducks. (*) denotes duck genes; (**) denotes chicken genes; no asterisks denote known orthologous genes in both species. The complete table, including SNP IDs, is available in Supplementary Tables S4 and S5.

	Effect	
**Gene**	**Duck**	**Chicken**
ENSAPLG00000013923 *, CD74 **	intron_variant	missense_variant, downstream_gene_variant
RUBCN	synonymous_variant	missense_variant
ABCA3	missense_variant	intron_variant
MAP2K6	missense_variant	intron_variant
VIPR2	missense_variant	intron_variant
ENSGALG00010000247 *, ENSAPLG00000008507 **	3_prime_UTR_variant, downstream_gene_variant, intron_variant	stop_gained, 3_prime_UTR_variant
SHTN1	intron_variant	splice_donor_variant, intron_variant
ATP8A1	splice_donor_variant, intron_variant	intron_variant
ENSAPLG00000022349 *, ERF3B **	splice_acceptor_variant, intron_variant	intron_variant

**Table 2:** Downstream pathways of the candidate gene lists in chickens and ducks.

Species	Pathway
Chicken	R-HSA-512988: Interleukin-3, Interleukin-5 and GM-CSF signaling
Duck	R-HSA-909733: Interferon alpha/beta signaling
	R-HSA-913531: Interferon Signaling
	R-HSA-1280215: Cytokine Signaling in Immune system
	R-HSA-168256: Immune System

**Table 3:** Downstream effectors of the candidate gene lists in chickens and ducks.

Chicken	Duck
IκBα	GSK-3β
axin1:β-catenin	ADRB2R:MBP
FAK1	ATF-2
β-catenin	STAT3
STAT1	IκBα
STAT3	STAT1
APC:axin1:β-catenin:GSK-3β	Tau (phosporylated)
-	APC:axin1:β-catenin:GSK-3β
-	c-Myc
-	p53
-	β-catenin
-	c-Jun
-	hist1h3f
-	Tau
-	EGFR

## Data Availability

Not applicable.

## Supplementary Materials

The following supporting information can be downloaded at: https://www.mdpi.com/article/10.3390/biology12070969/s1, Figure S1: downstream effectors of chicken; Figure S2: downstream effectors of duck; File S1: source code of the workflow for coSNP identification and analysis; File S2: list of chicken orthologs of duck DEGs that are affected by a coSNP; File S3: list of duck DEGs that are affected by a coSNP; Table S1: overview of all identified coSNPs; Table S2: overview of the number of coSNP impacts; Table S3: overview of DEG expression; Table S4: summary of impacts (moderate or high) of coSNPs on chicken orthologs of duck DEGs; Table S5: summary of impacts (moderate or high) of coSNPs on duck DEGs; Table S6: identified effectors of chicken orthologs of duck DEGs impacted (moderate or high) by a coSNP; Table S7: identified effectors of duck DEGs impacted (moderate or high) by a coSNP; Table S8: Reactome enriched pathways of chicken orthologs of duck DEGs impacted (moderate or high) by a coSNP; Table S9: Reactome enriched pathways of duck DEGs impacted (moderate or high) by a coSNP.

## Abbreviations

The following abbreviations are used in this manuscript:

AIV	avian influenza virus
coSNP	coincident SNP
DEG	differentially expressed gene
dpi	days post infection
FAK1	focal adhesion kinase 1
FDR	false discovery rate
GM-CSF	granulocyte-macrophage colony-stimulating factor
HPAIV	highly pathogenic AIV
IFN	interferon
IL	interleukin
ISG	IFN-stimulated gene
JNK	c-Jun N-terminal kinase
LPAIV	low pathogenic AIV
PI3K	phosphoinositide 3-kinase
PRR	pattern recognition receptor
RIG-I	retinoic acid inducible gene I
RLR	RIG-I-like receptor
SNP	single-nucleotide polymorphism
STAT	signal transducer and activator of transcription family
TF	transcription factor
TRIM	tripartite motif
