# Supplementary material for: Comparative Investigation of Coincident Single Nucleotide Polymorphisms Underlying Avian Influenza Viruses in Chickens and Ducks
Source: Biology (Basel). 2023 Jul 7;12(7):969. doi: 10.3390/biology12070969 (PMC10375970; doi:10.3390/biology12070969)
Supplement: Supplementary file 1 [file biology-12-00969-s001.zip › supplementary_files/supplementary_fig_S2_duck_effectors.pdf]

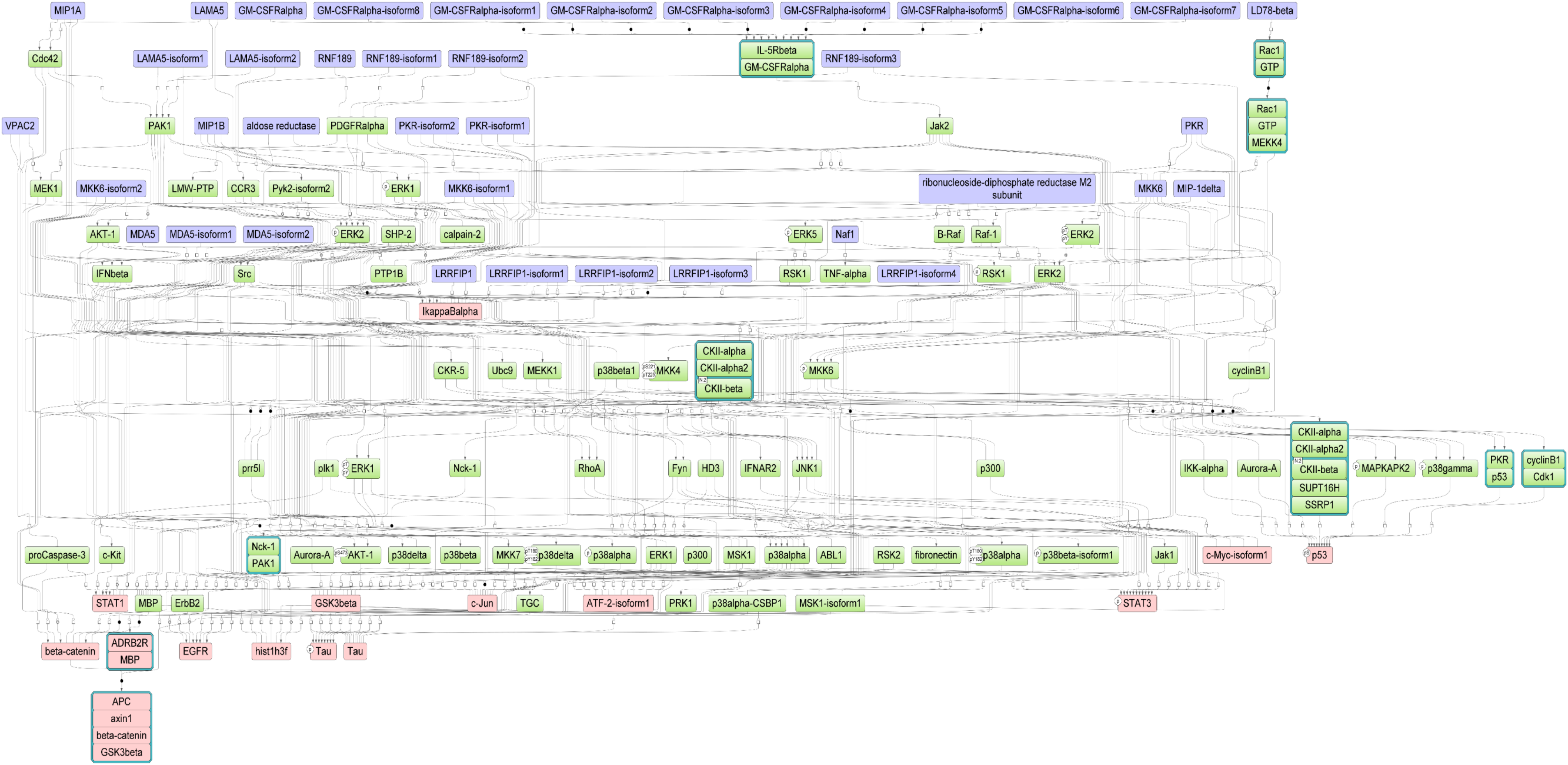

Figure S2: Overview of identified TRANSPATH downstream effectors and their interactions corresponding to the **duck** gene list. Blue nodes mark the input genes, green nodes are intermediate interaction partners and red nodes represent the identified downstream effectors. Each edge relates to a curated interaction retrieved from the TRANSPATH database.
