# Supplementary material for: Comparative Investigation of Coincident Single Nucleotide Polymorphisms Underlying Avian Influenza Viruses in Chickens and Ducks
Source: Biology (Basel). 2023 Jul 7;12(7):969. doi: 10.3390/biology12070969 (PMC10375970; doi:10.3390/biology12070969)
Supplement: Supplementary file 1 [file biology-12-00969-s001.zip › supplementary_files/supplementary_file_S1_source_code.zip!/supplementary_S1_source_code/analysis.pdf]

Workflow coSNP Analysis

2023-02-14

coSNP Analysis

First, coSNPs are identified with the workflow described in the paper using a liftover pipeline. Results are available in the supplementary files.

Libraries

```
library(tidyverse)
```

```
## -- Attaching packages --------------------------------------- tidyverse 1.3.2 --
## v ggplot2 3.4.1     v purrr   1.0.1
## v tibble  3.1.8     v dplyr   1.1.0
## v tidyr   1.3.0     v stringr 1.5.0
## v readr   2.1.4     v forcats 1.0.0
## -- Conflicts ------------------------------------------ tidyverse_conflicts() --
## x dplyr::filter() masks stats::filter()
## x dplyr::lag()    masks stats::lag()
```

```
library(gprofiler2)
```

Session Info

In case some issues arise due to incompatible R package versions.

```
sessionInfo()
```

```
## R version 4.2.2 Patched (2022-11-10 r83330)
## Platform: x86_64-pc-linux-gnu (64-bit)
## Running under: Linux Mint 21
##
## Matrix products: default
## BLAS:   /usr/lib/x86_64-linux-gnu/blas/libblas.so.3.10.0
## LAPACK: /usr/lib/x86_64-linux-gnu/lapack/liblapack.so.3.10.0
##
## locale:
##  [1] LC_CTYPE=de_DE.UTF-8       LC_NUMERIC=C
##  [3] LC_TIME=de_DE.UTF-8        LC_COLLATE=de_DE.UTF-8
##  [5] LC_MONETARY=de_DE.UTF-8    LC_MESSAGES=de_DE.UTF-8
##  [7] LC_PAPER=de_DE.UTF-8       LC_NAME=C
##  [9] LC_ADDRESS=C               LC_TELEPHONE=C
## [11] LC_MEASUREMENT=de_DE.UTF-8 LC_IDENTIFICATION=C
##
## attached base packages:
## [1] stats     graphics  grDevices utils     datasets  methods   base
##
## other attached packages:
```

```
##  [1] gprofiler2_0.2.1 forcats_1.0.0    stringr_1.5.0    dplyr_1.1.0
##  [5] purrr_1.0.1      readr_2.1.4      tidyr_1.3.0      tibble_3.1.8
##  [9] ggplot2_3.4.1    tidyverse_1.3.2
##
## loaded via a namespace (and not attached):
##  [1] tidyselect_1.2.0    xfun_0.37           haven_2.5.1
##  [4] gargle_1.3.0        colorspace_2.1-0    vctrs_0.5.2
##  [7] generics_0.1.3      viridisLite_0.4.1   htmltools_0.5.4
## [10] yaml_2.3.7          plotly_4.10.1       utf8_1.2.3
## [13] rlang_1.0.6         pillar_1.8.1        glue_1.6.2
## [16] withr_2.5.0         DBI_1.1.3           dbplyr_2.3.0
## [19] modelr_0.1.10       readxl_1.4.2        lifecycle_1.0.3
## [22] munsell_0.5.0       gtable_0.3.1        cellranger_1.1.0
## [25] rvest_1.0.3         htmlwidgets_1.6.1   evaluate_0.20
## [28] knitr_1.42          tzdb_0.3.0          fastmap_1.1.0
## [31] fansi_1.0.4         broom_1.0.3         scales_1.2.1
## [34] backports_1.4.1     googlesheets4_1.0.1 jsonlite_1.8.4
## [37] fs_1.6.1            hms_1.1.2           digest_0.6.31
## [40] stringi_1.7.12      grid_4.2.2          cli_3.6.0
## [43] tools_4.2.2         magrittr_2.0.3      lazyeval_0.2.2
## [46] crayon_1.5.2        pkgconfig_2.0.3     ellipsis_0.3.2
## [49] data.table_1.14.8   xml2_1.3.3          reprex_2.0.2
## [52] googledrive_2.0.0   lubridate_1.9.2     timechange_0.2.0
## [55] assertthat_0.2.1    rmarkdown_2.20      httr_1.4.4
## [58] rstudioapi_0.14     R6_2.5.1            compiler_4.2.2
```

Function Definitions

For reading in `gff3` and `gvf` data:

```
gff3Format <- function(file) {
  if (!file.exists(file)) {
    msg <- paste0("file ", file, " does not exist")
    stop(msg)
  }

  # Read in gff3 line by line and discard headers containing #
  gff3 <- readLines(file)
  gff3 <- gff3[!startsWith(gff3, "#")]
  str <- strsplit(gff3, "\t")

  # Filter rows that do not contain the "gene" identifier in the third column
  gff3 <- str[sapply(str, \(x) x[3] == "gene")] # to also include non-coding genes: grepl("gene", x[3])

  # Retrieve information from the columns of the remaining file
  info <- sapply(gff3, \(x) x[9])
  id <- unlist(gsub(".*;gene_id=(\\w+);.*", "\\1", info))
  chrom <- sapply(gff3, \(x) x[1])
  strand <- sapply(gff3, \(x) x[7])
  start <- sapply(gff3, \(x) x[4])
  end <- sapply(gff3, \(x) x[5])

  data.frame(
    gene = id,
```

```
    chr = chrom,
    strand = as.character(strand),
    start = as.numeric(start),
    end = as.numeric(end)
  )
}

gvfFormat <- function(file) {
  if (!file.exists(file)) {
    msg <- paste0("file ", file, " does not exist")
    stop(msg)
  }

  # Read in file, retrieve information and return data.frame
  readr::read_delim(file,
                    comment = '#',
                    col_names = FALSE,
                    delim = '\t',
                    show_col_types = FALSE) %>%
    # Ignore 5th line since only SNPs are retrieved
    dplyr::select(-5) %>%
    dplyr::rename(
      chr = X1,
      source = X2,
      type = X3,
      POS = X4,
      QUAL = X6,
      strand = X7,
      FILTER = X8,
      INFO = X9
    ) %>%
    dplyr::filter(type == "SNV") %>% # filter for SNPs
    # Retrieve SNP ID from INFO column after Dbxref=
    dplyr::mutate(ID = stringr::str_replace(INFO, ".*Dbxref=[[:alnum:]|_]*:(\\w+);.*", "\\1")) %>%
    # Retrieve SNP reference allele from INFO column after Reference_seq=
    dplyr::mutate(REF = stringr::str_replace(INFO, ".*Reference_seq=(\\w).*", "\\1")) %>%
    # Retrieve SNP alternative allele from INFO column after Variant_seq=
    dplyr::mutate(ALT = stringr::str_replace(INFO, ".*Variant_seq=(\\w).*", "\\1")) %>%
    dplyr::select(chr, POS, ID, REF, ALT, QUAL, FILTER, INFO)
}
```

Reading in files

Suppose all of the files are in `data/`.

Required files:

- identified cosnps (here: `cosnp_cosnps.csv`)
- `gff3` files from Ensembl Release 109 for `CAU_duck1.0` and `GRCg7b` (here: `duck.gff3`, `chicken.gff3`)
- `gvf` variant file from Ensembl Release 109 for `GRCg7b` (here: `chicken.gvf`)
- mapped variant file for `CAU_duck1.0` (here: `liftover_mapped.vcf`)

This may take a while. May require > 15GB of RAM.

```r
# workflow output
cosnps <- read_csv("data/cosnp_cosnps.csv", show_col_types = FALSE)

# gene files
chicken_genes <- gff3Format("data/chicken.gff3") %>%
  tibble()
duck_genes <- gff3Format("data/duck_cau.gff3") %>%
  tibble()
# variant files
chicken_snps <- gvfFormat("data/chicken.gvf") %>%
  tibble()
duck_snps <- read_delim("data/liftover_mapped.vcf", delim = "\t", comment = "##", col_names = TRUE, sho
```

Merge chicken and duck SNP and coSNP tables

```r
duck_cosnps <- cosnps %>%
  distinct(ID_query) %>%
  inner_join(duck_snps, by = c("ID_query" = "ID"))

chicken_cosnps <- cosnps %>%
  distinct(ID_target) %>%
  mutate(ID_target = sapply(strsplit(ID_target, ":"), \(x) x[2])) %>%
  inner_join(chicken_snps, by = c("ID_target" = "ID"))
```

Identify coSNPs located in genes

```r
# cosnps in duck/chicken genes
duck_cosnps_genes <- duck_cosnps %>%
  inner_join(duck_genes, by = c("#CHROM" = "chr"), multiple = "all") %>%
  filter(POS >= start & POS <= end)

chicken_cosnps_genes <- chicken_cosnps %>%
  inner_join(chicken_genes, by = c("chr"), multiple = "all") %>%
  filter(POS >= start & POS <= end)

# merged result table
all_cosnps_genes <- cosnps %>%
  inner_join(
    duck_cosnps_genes,
    by = c(
      "CHROM" = "#CHROM",
      "ID_query",
      "REF_query" = "REF",
      "ALT_query" = "ALT",
      "POS"
    ),
    multiple = "all"
  ) %>%
  select(-c(QUAL, FILTER, INFO)) %>%
  mutate(ID_target = sapply(strsplit(ID_target, ":"), \(x) x[2])) %>%
  inner_join(
    chicken_cosnps_genes,
```

```
    by = c("ID_target"),
    suffix = c("_query", "_target"),
    multiple = "all"
  ) %>%
  select(-c(QUAL, FILTER, INFO))
all_cosnps_genes
```

```
## # A tibble: 39,734 x 20
##    ID_ta~1 CHROM POS_q~2 REF_t~3 ALT_t~4 ID_qu~5 REF_q~6 ALT_q~7 gene_~8 stran~9
##    <chr>   <chr>   <dbl> <chr>   <chr>   <chr>   <chr>   <chr>   <chr>   <chr>
##  1 rs3179~ 1       23108 C       A       apl266~ T       A       ENSAPL~ -
##  2 rs1060~ 1       31986 G       A       apl266~ C       T       ENSAPL~ -
##  3 rs1380~ 1       31991 G       A       apl266~ G       C       ENSAPL~ -
##  4 rs1059~ 1       32860 G       A       apl266~ A       C       ENSAPL~ -
##  5 rs1058~ 1       38686 G       T       apl266~ C       T       ENSAPL~ -
##  6 rs1059~ 1       40040 G       A       apl266~ T       G       ENSAPL~ -
##  7 rs1058~ 1       66842 G       A       apl266~ C       G       ENSAPL~ -
##  8 rs7334~ 1       67463 A       C       apl266~ C       A       ENSAPL~ -
##  9 rs3134~ 1       74240 T       C       apl266~ G       T       ENSAPL~ -
## 10 rs3181~ 1       88177 G       C       apl266~ A       G       ENSAPL~ -
## # ... with 39,724 more rows, 10 more variables: start_query <dbl>,
## #   end_query <dbl>, chr <chr>, POS_target <dbl>, REF <chr>, ALT <chr>,
## #   gene_target <chr>, strand_target <chr>, start_target <dbl>,
## #   end_target <dbl>, and abbreviated variable names 1: ID_target,
## #   2: POS_query, 3: REF_target, 4: ALT_target, 5: ID_query, 6: REF_query,
## #   7: ALT_query, 8: gene_query, 9: strand_query
```

`all_cosnps_genes` now contains the contrast of chicken and duck coSNPs.

Write back to `vcf` files:

```
# duck
duck_vcf_cosnps <- duck_snps %>%
  filter(ID %in% all_cosnps_genes$ID_query)

write_delim(duck_vcf_cosnps,
            "data/duck_cosnps.vcf",
            delim = "\t")

# chicken
chicken_vcf_cosnps <- chicken_snps %>%
  filter(ID %in% all_cosnps_genes$ID_target) %>%
  dplyr::rename(`#CHROM` = chr)

write_delim(
  chicken_vcf_cosnps,
  "data/chicken_cosnps.vcf",
  delim = "\t")
```

Effect of SNPs

Calculate the effect of coSNPs with `SnpEff` (assuming jar is in `snpEff/` directory):

- Prerequisite: Create `SnpEff` databases for `Ensembl Release 109` genomes `CAU_duck1.0` and `GRCg7b` (based on available toplevel assembly and `gtf` file).

```r
system2(
  "java",
  c(
    "-jar",
    "-Xmx16g",
    "snpEff/snpEff.jar",
    "CAU_duck1.0.109",
    "data/duck_cosnps.vcf",
    "-csvStats",
    "data/duck_cosnps_eff.csv"
  ),
  "data/duck_cosnps.ann.vcf"
)
system2(
  "java",
  c(
    "-jar",
    "-Xmx16g",
    "snpEff/snpEff.jar",
    "GRCg7b.109",
    "data/chicken_cosnps.vcf",
    "-csvStats",
    "data/chicken_cosnps_eff.csv"
  ),
  stdout = "data/chicken_cosnps.ann.vcf"
)
```

Plot general trend of SNP effects on genes

```r
# Read in and summarise effect frequencies
snpeff_chicken <-
  read_delim(
    "data/chicken_cosnps_eff.genes.txt",
    delim = "\t",
    comment = "# ",
    col_names = TRUE,
    show_col_types = FALSE
  )

snpeff_duck <-
  read_delim(
    "data/duck_cosnps_eff.genes.txt",
    delim = "\t",
    comment = "# ",
    col_names = TRUE,
    show_col_types = FALSE
  )

get_impact_frequencies <- function(snpeff_chicken, snpeff_duck) {
    chicken_freqs <- snpeff_chicken %>%
  summarise(across(5:ncol(.), sum)) %>%
  pivot_longer(cols = 1:ncol(.))
```

```r
    duck_freqs <- snpeff_duck %>%
      summarise(across(5:ncol(.), sum)) %>%
      pivot_longer(cols = 1:ncol(.))

    # frequencies in long format
    freqs <- bind_rows(duck_freqs, chicken_freqs) %>%
      mutate(species = c(rep("duck", nrow(.) / 2), rep("chicken", nrow(.) / 2)))

    # table overview
    overview <- inner_join(duck_freqs, chicken_freqs, by = "name") %>%
      dplyr::rename(effect = name,
                    duck = value.x,
                    chicken = value.y)
    return(list(freqs = freqs, overview = overview))
}

freq_list <- get_impact_frequencies(snpeff_chicken, snpeff_duck)

# comparative plot of frequencies
ggplot(freq_list$freqs) +
  geom_bar(aes(x = name, y = value, fill = species), stat = "identity") +
  theme(axis.text.x = element_text(
    angle = 90,
    vjust = 0.5,
    hjust = 1,
    size = 5
  )) +
  labs(x = "Effect", y = "Count")
```

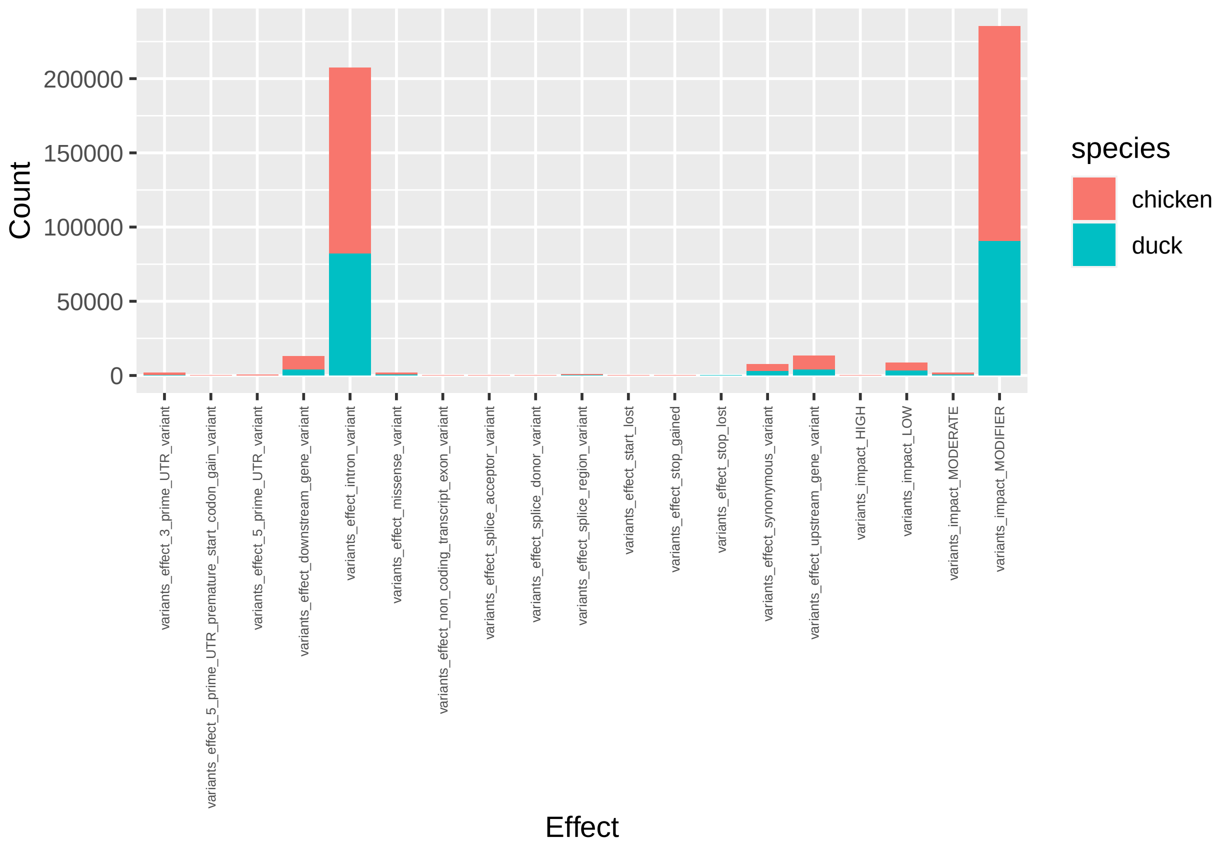


```r
write_csv(freq_list$overview, "data/impact_overview.csv")
freq_list$overview
```

```
## # A tibble: 17 x 3
##    effect                                                     duck chicken
##    <chr>                                                     <dbl>   <dbl>
##  1 variants_impact_HIGH                                         23      13
##  2 variants_impact_LOW                                        3446    5115
##  3 variants_impact_MODERATE                                    703    1254
##  4 variants_impact_MODIFIER                                  90685  144735
##  5 variants_effect_3_prime_UTR_variant                         505    1481
##  6 variants_effect_5_prime_UTR_premature_start_codon_gain_variant   28      63
##  7 variants_effect_5_prime_UTR_variant                         168     370
##  8 variants_effect_downstream_gene_variant                    3898    9055
##  9 variants_effect_intron_variant                            82237  125117
## 10 variants_effect_missense_variant                            703    1254
## 11 variants_effect_splice_acceptor_variant                       3       1
## 12 variants_effect_splice_donor_variant                          4       4
## 13 variants_effect_splice_region_variant                       377     536
## 14 variants_effect_start_lost                                    1       1
## 15 variants_effect_stop_gained                                  11       7
## 16 variants_effect_synonymous_variant                         3105    4599
## 17 variants_effect_upstream_gene_variant                      4194    9163
```

Genes with high or moderate impact

```
impacted_genes_chicken <- snpeff_chicken %>%
  filter(variants_impact_HIGH > 0 | variants_impact_MODERATE > 0)

impacted_genes_duck <- snpeff_duck %>%
  filter(variants_impact_HIGH > 0 | variants_impact_MODERATE > 0)

# frequencies for high/moderare impact genes
freq_list <- get_impact_frequencies(impacted_genes_chicken, impacted_genes_duck)

# comparative plot of frequencies
ggplot(freq_list$freqs) +
  geom_bar(aes(x = name, y = value, fill = species), stat = "identity") +
  theme(axis.text.x = element_text(
    angle = 90,
    vjust = 0.5,
    hjust = 1,
    size = 5
  )) +
  labs(x = "Effect", y = "Count")
```

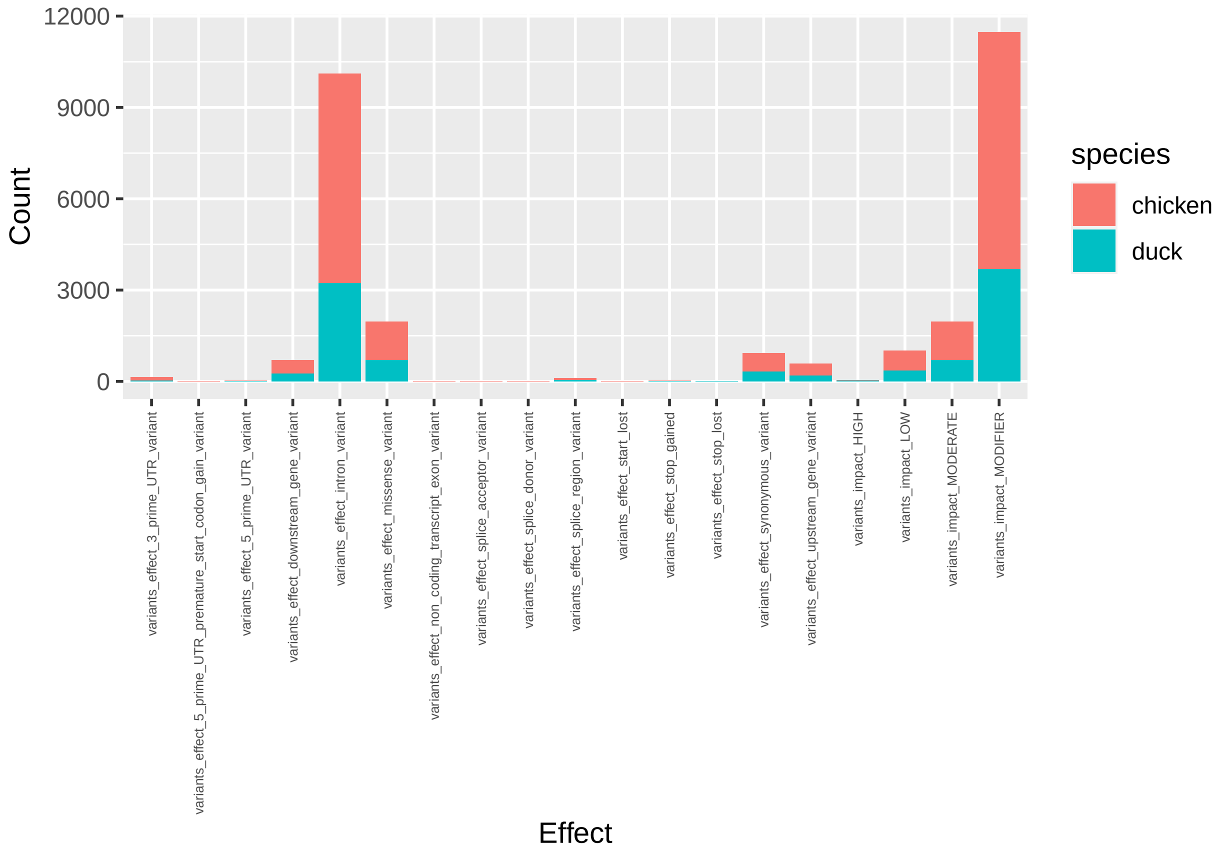


```
write_csv(freq_list$overview, "data/high_impact_overview.csv")
freq_list$overview
```

```
## # A tibble: 17 x 3
##    effect                                                           duck chicken
```

```
##    <chr>                                                        <dbl>   <dbl>
##  1 variants_impact_HIGH                                            23      13
##  2 variants_impact_LOW                                            364     641
##  3 variants_impact_MODERATE                                       703    1254
##  4 variants_impact_MODIFIER                                      3698    7781
##  5 variants_effect_3_prime_UTR_variant                             30     114
##  6 variants_effect_5_prime_UTR_premature_start_codon_gain_variant   3       1
##  7 variants_effect_5_prime_UTR_variant                              8      11
##  8 variants_effect_downstream_gene_variant                        261     443
##  9 variants_effect_intron_variant                                3235    6868
## 10 variants_effect_missense_variant                               703    1254
## 11 variants_effect_splice_acceptor_variant                          3       1
## 12 variants_effect_splice_donor_variant                             4       4
## 13 variants_effect_splice_region_variant                           48      60
## 14 variants_effect_start_lost                                       1       1
## 15 variants_effect_stop_gained                                     11       7
## 16 variants_effect_synonymous_variant                             332     603
## 17 variants_effect_upstream_gene_variant                          200     387
```

DEGs of duck and orthologs in chicken with high or moderate impact

Requires DEG files from previous study: https://doi.org/10.3390/biology11020219

Can be downloaded from: https://github.com/SelinaKlees/AIV_chicken_duck

```r
# read in DEGs of duck
deg_sets <-
  file.path(
    "data/AIV_chicken_duck/Duck/DEG/",
    dir("data/AIV_chicken_duck/Duck/DEG/", pattern = "H5.*.txt")
  )
deg_list <-
  lapply(deg_sets,
         \(x) read_delim(x, delim = "\t", show_col_types = FALSE) %>% pull(gene)) %>%
  unlist() %>%
  unique()
# orthologs in chicken
orth_list <-
  gorth(deg_list,
        source_organism = "applatyrhynchos",
        target_organism = "ggallus") %>%
  pull(ortholog_ensg) %>%
  unique()

# duck DEGs with coSNP effect
filter_degs <- function(impacted_genes, degs) {
  impacted_degs <- impacted_genes %>%
    filter(GeneId %in% degs) %>%
    pull(GeneId) %>%
    unique()
  return(impacted_degs)
}

impacted_degs_duck <- filter_degs(impacted_genes_duck, deg_list)
impacted_degs_chicken <- filter_degs(impacted_genes_chicken, orth_list)
```

```
# write files to disk
write_lines(impacted_degs_duck, "data/impacted_degs_duck.txt")
write_lines(impacted_degs_chicken, "data/impacted_degs_chicken.txt")
```

Note that gprofiler may map orthologs differently in future releases.

The two text files `impacted_degs_duck.txt` and `impacted_degs_chicken.txt` are the main gene lists that are further analysed.

Downstream Effectors

Downstream effectors of the target gene lists are separately determined by TRANSPATH provided by the genexplain platform. An overview of the workflow may be found here.

A maximum radius of 10 was applied and effectors are filtered based on the default values for score, FDR and z-score.

Results of this step are also provided in the supplementary files.
